# Site-specific incubations reveal biofilm diversity and functional adaptations in deep, ancient desert aquifers

**DOI:** 10.3389/fmicb.2025.1533115

**Published:** 2025-03-21

**Authors:** Betzabe Atencio, Stas Malavin, Maxim Rubin-Blum, Roi Ram, Eilon Adar, Zeev Ronen

**Affiliations:** ^1^Department of Environmental Hydrology and Microbiology, Zuckerberg Institute for Water Research, Ben-Gurion University of the Negev, Sede Boqer Campus, Israel; ^2^Department of Marine Biology, Israel Oceanographic and Limnological Research, Haifa, Israel; ^3^Department of Marine Biology, Leon H. Charney School of Marine Sciences, University of Haifa, Haifa, Israel; ^4^Geological Survey of Israel, Jerusalem, Israel; ^5^Institute of Environmental Physics, Heidelberg University, Heidelberg, Germany

**Keywords:** deep subsurface, deep aquifers, groundwater, attached-sessile communities, biofilm

## Abstract

Deep pristine aquifers are ecological hotspots with diverse microbial life, where microorganisms exist either attached (sessile) to solid substrates or suspended in groundwater (planktonic). Characterizing the attached microbial communities is of paramount importance, especially in the context of biofouling. However, obtaining samples of attached microbes that thrive under natural (undisturbed) conditions is challenging. Our study addresses this by retrieving sessile microbes on-site. We installed columns filled with site-specific rock cuttings at the wellhead, allowing fresh groundwater to flow continuously for approximately 60 days. We hypothesized that the attached microbial communities would differ structurally from planktonic microbes due to the aquifer’s lithological and mineralogical composition. This study involved an exploratory examination of the microbial communities in different aquifers with distinct mineralogies, including quartzitic sandstone, calcareous, chert, and highly heterogeneous (clastic) aquifers in Israel’s Negev Desert. Metagenomic analysis revealed both shared and distinct microbial communities among attached and planktonic forms in the various environments, likely shaped by the aquifers’ physical, lithological, and mineralogical properties. A wealth of carbon-fixation pathways and energy-conservation strategies in the attached microbiome provide evidence for the potential productivity of these biofilms. We identified widespread genetic potential for biofilm formation (e.g., via pili, flagella, and extracellular polymeric substance production) and the interactome (e.g., quorum-sensing genes). Our assessment of these functions provides a genomic framework for groundwater management and biofouling treatment.

## 1 Introduction

Groundwater, often hidden in aquifers, is one of the most significant freshwater reserves on Earth, representing around 30% of global freshwater resources—far exceeding the combined volume of all lakes, rivers, and atmospheric water (Shiklomanov, 1993). Beneath the surface, aquifers not only store vital water resources but also support vast microbial communities. Recent estimates suggest that bacteria form the second-largest reservoir of biomass on Earth, approximately 70 gigatons of carbon, and that subsurface environments host most of the bacterial and archaeal biomass (Bar-On et al., 2018). This deep subsurface biomass accounts for about 15% of the Earth’s total biomass and is largely composed of surface-attached bacteria and archaea thriving within the aquifers (Bar-On et al., 2018).

In aquifers, microbial communities exist in two distinct states: free-living planktonic microbes suspended in the groundwater and sessile microbes attached to the solid surfaces of the aquifer matrix, forming biofilms (Smith et al., 2018). Attached microbial communities in aquifers are thought to harbor up to two magnitudes greater microbial mass than those suspended in the groundwater, indicating their potentially crucial role in subsurface ecosystems (Griebler and Lueders, 2009; Casar et al., 2021; Gios et al., 2023). The aquifer matrix’s mineralogical characteristics affect these microbial communities’ structure and function. For instance, specific minerals can promote selective colonization by microbes, driving variations in community composition and functional potential (Mullin et al., 2020; Casar et al., 2021). Similar dynamics have been observed in subsurface environments globally, where attached and planktonic microbial communities demonstrate metabolic versatility and play critical roles in biogeochemical cycles under redox-dynamic conditions (Magnabosco et al., 2018; Mullin et al., 2020; Dong et al., 2021; Meyer-Dombard and Malas, 2022). Similar to aquifers, microbial communities adapt to oligotrophic conditions and fluctuating redox conditions in subsurface coal seams, engaging in carbon cycling, nitrogen fixation, and hydrocarbon degradation (Vick et al., 2019). Such communities are shaped by environmental parameters such as minerals concentrations, gas composition, and redox gradients, which influence microbial partitioning between planktonic and attached fractions (Zhang et al., 2024).

The attached sessile microbes often demonstrate significant differences in composition, function, and productivity compared to their planktonic counterparts (Lehman et al., 2001; Flynn et al., 2008, 2013; Nuppunen-Puputti et al., 2020; Dong et al., 2021; Meyer et al., 2022; Meyer-Dombard and Malas, 2022). However, their adhesion to the aquifer matrix and difficulty accessing deep subsurface environments make obtaining and studying these attached bacteria challenging. Novel methods, such as exposure of different substrates to groundwater and the use of flow-cell cabinets that simulate hard rock aquifers, have been developed to investigate these communities (Hallbeck and Pedersen, 2008; Griebler and Lueders, 2009; Wu et al., 2017; Lopez-Fernandez et al., 2023).

While our knowledge of planktonic microbes has expanded through the study of water samples extracted from deep fractures and aquifers, the attached microbial communities remain less understood. Much remains to be explored regarding the compositional differences between attached and suspended microbial communities and their respective roles in biogeochemical cycles. For instance, in aquifers in regions such as South Africa, east central Illinois in the US, and the Canadian Shield, attached microbes have been shown to contribute disproportionately to processes such as carbon fixation, nitrogen cycling, and methane oxidation (Blanco et al., 2014; Ruff et al., 2023; Flynn et al., 2008). These findings provide valuable parallels for understanding the critical functions of microbial communities in the aquifers of arid and hyperarid regions.

In arid and hyperarid regions, such as Israel’s Arava and Negev deserts, ancient brackish groundwater from deep aquifers, such as the Nubian Sandstone Aquifer (NSA), has become a critical resource for domestic water supply and agricultural irrigation (Ghermandi and Messalem, 2009). Boreholes in these regions often reach depths exceeding 1 km, tapping into these ancient aquifers. A large agricultural industry has developed in these regions, relying on reverse-osmosis desalination of brackish groundwater abstracted from deep sandstone and calcareous aquifers. Despite the overall effectiveness of these desalination systems, they often encounter biofouling challenges, likely driven by native microbes in the aquifers (Gino et al., 2010; Sweity et al., 2015). These types of processes have been documented in groundwater systems in Israel, where biofouling and biofilm-associated corrosion have been linked to the activities of iron-reducing and sulfur-oxidizing bacteria (*Gallionella, Thiobacillus, Leptothrix, Sphaerotilus*), as well as sulfate-reducing bacteria (SRB) (*Desulfovibrio, Desulfotomaculum*) (Laor, 2015).

Our previous study identified planktonic microbial communities in the NSA and the overlying Judea Group Carbonate Aquifer (JCA) that appear to play roles in key biogeochemical cycles, such as the carbon, nitrogen, sulfur, and hydrogen cycles (Atencio et al., 2024). However, attached microbial communities, which are likely influenced by the aquifers’ lithological, mineralogical, and redox conditions, may play an equally important role. For example, the NSA and JCA, which are primarily composed of quartzitic sandstone and calcareous limestone, contain ancient groundwater. However, their distinct lithologies create varied geochemical conditions that influence microbial community structure and biogeochemical processes. One sampling site shows signs of intrusion from a brackish deep aquifer (BDA), which may introduce additional chemical and microbial variability.

Furthermore, other local aquifers, such as the chert aquifer [Senonian Chalk & Chert Mishash Formation (SCA)] and highly heterogeneous (clastic) aquifers [Arava Fill and Hazeva Formation aquifers (AFA)], also provide interesting comparisons. These aquifers share a similar recharge mechanism via floodwater percolation, coupled with their proximity to recharge zones (Ram et al., 2021); however, they exhibit distinct geochemical characteristics, including differences in oxygen availability and mineral content, which could impact microbial community structure and biogeochemical processes, such as sulfur cycling (Supplementary Tables ST1, ST2; Supplementary Figures S1, S2).

We hypothesize that the attached microbial communities in key aquifers of the Arava Valley and Negev Desert, shaped by distinct lithologies and redox conditions, play a crucial role in biogeochemical processes such as sulfur cycling and carbon fixation. These microbial communities are likely to impact water-treatment systems by forming biofilms, which could influence system efficiency and long-term operational stability. This study aims to (i) analyze and compare the compositional differences between planktonic microbial communities and those attached to the subsurface aquifer matrix, (ii) understand the potential interactions between populations and the influence of environmental factors on these communities, and (iii) investigate the potential functional role of these communities in biogeochemical cycles.

To achieve this, we employed a custom-made filtration system using well-documented rock cuttings from the National Geological Archive of the Geological Survey of Israel, allowing us to reconstruct the in-situ microenvironment for substrate-attached microbial communities and provide a comprehensive view of their role in the studied deep aquifers. This study’s wells’ depths range from approximately 200 to 1,500 meters below the surface, tapping into multiple aquifers that exhibit different geochemical profiles.

## 2 Materials and methods

### 2.1 Sample collection

**Figure 1:**
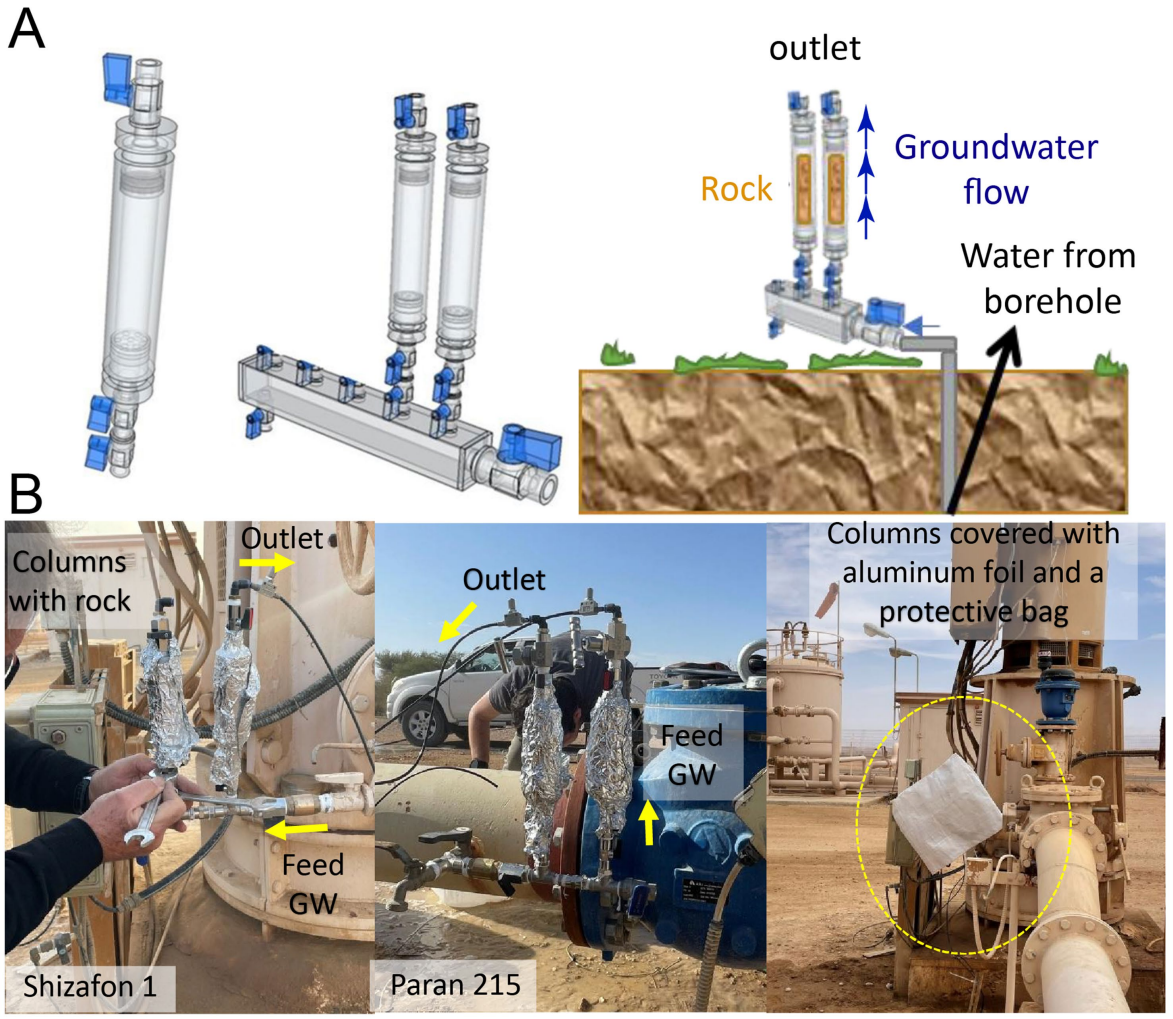
Design of in-situ microbial inoculation bypass column flow cells (Type 1) **(A)** In-situ incubation in the field, where rock cuttings inside the columns were exposed to groundwater (GW) fluxes in production wells **(B)**.

To better understand the native microbial communities in the aquifers, we sampled sessile (attached) and planktonic (suspended) microorganisms. We used the approach outlined by Atencio et al. (2024) to evaluate planktonic microbes. We employed a passive sampling method, simulating the aquifer matrix, to study the sessile/attached microorganisms (Figure 1). This method involved exposing a mixture of the original rock cuttings collected from segments of subaquifer units to fresh pumped groundwater in in-situ column-bypass flow cells mounted in each of the selected production wells. Rock cuttings were obtained from every aquifer water-bearing unit following the stratigraphic borehole logs and technical specifications of each of the chosen production wells. As each segment in front of the filters contributes groundwater to the well but does not have the physical properties of each layer, we could not estimate the relative contribution of the water-bearing formations to the volume pumped from the aquifer. Given that these production wells fed through several screens, we mixed rock cuttings only from those productive aquifer segments (Supplementary Table ST1; Supplementary Figure S1).

Each microbial trap was constructed with a column cell using cylindrical polyvinylidene fluoride (PVDF) material, which is characterized by high thermal stability and is chemically inert, with dimensions of 5.5 cm in length and outer and inner diameters of 4 cm and 2.5 cm, respectively (Figure 1). A 50-μm mesh polyester bag was filled with 25 g of the respective homogenized rock cuttings and placed inside the PVDF cells with glass beads filling the extra space. Each cell was equipped with double-needle valves on each side and two non-return valves installed at the inlet. The rock cuttings in the mesh bags were sequentially washed with sterile Milli-Q water and sterilized. Following column assembly and loading of the rock cuttings, the entire setup, including the connectors and manifold, was subjected to double sterilization.

To avoid interfering with the high pumping rates of the production wells, the column cells were mounted on a 0.5-inch bypass equipped with two non-return valves. Since these production wells stop pumping when the storage system is full or the water consumption decreases, the non-return valves prevented the water from being sucked back into the well. Thus, even if the production wells temporarily ceased operation, the material in the column remained fully saturated with water until pumping resumed. This setup provided continuous contact of the rock sediments with fresh pumped groundwater. The delicate needle valves allowed adjustment and controlled a steady, slow flow, similar to groundwater flow velocities in the aquifer, on the order of 1 m per year. The flow rates through the columns were adjusted to about 0.5 L per day; the column cells are prone to clogging at a slower rate. The columns were protected with layers of tin foil and a covering bag to prevent any effects of sunlight.

**Figure 2:**
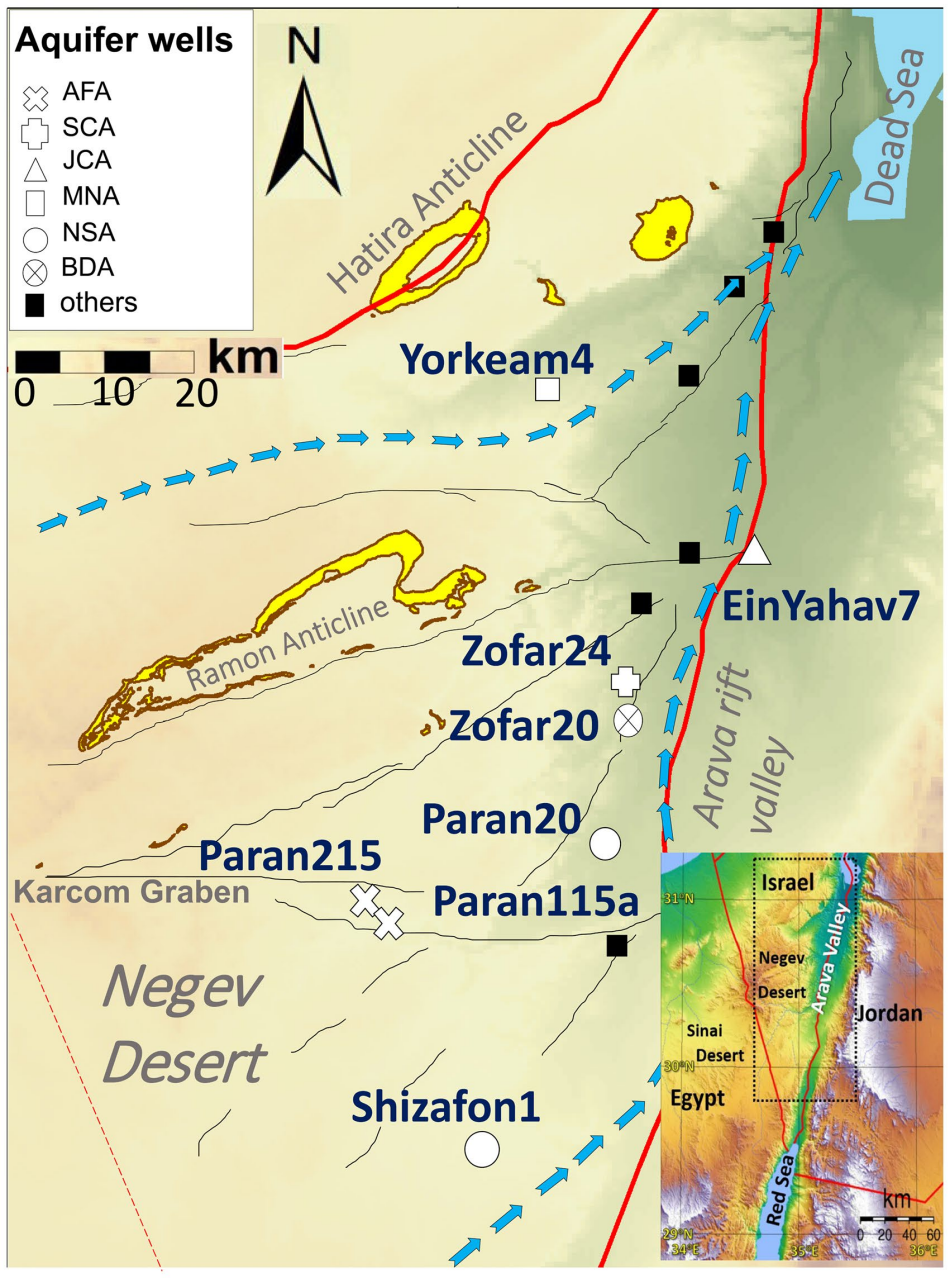
Location of sampled wells containing planktonic and attached communities. AFA, Arava Fill and Hazeva aquifers (highly heterogeneous clastic material); SCA, Senonian Chalk and Chert (Mishash formation: cherts, phosphorites, organic-rich carbonates); JCA, Judea Carbonate Group Aquifer (limestone/calcareous); MNA, mixed younger Nubian Sandstone Aquifer (quartzitic sandstone); NSA, Nubian Sandstone Aquifer (quartzitic sandstone); BDA, Brackish Deep Aquifer (quartzitic sandstone). Blue arrows indicate main flow directions in the Negev Desert regional aquifers (modified after Ram et al., 2020).

We conducted field experiments at multiple well sites in the Arava Valley and Negev Desert between October 2021 and November 2022 (Figure 2). The experiment involved two columns per well site, with incubation periods of 60 and 115 days, respectively, positioned at the wellheads to capture microbial communities directly from the groundwater before any atmospheric exposure or treatment. Although these on-site conditions do not fully replicate the pressure and temperature of the deep aquifers, the columns provided valuable insights into the attached microbial communities. At the end of the incubation, the column microbial traps were immediately placed in sterile bags, frozen using dry ice, and transported on the same day to the laboratory. Upon returning to the Ben Gurion University of the Negev laboratory, the columns were disassembled inside an anaerobic hood (Coy Laboratory Products) and the rock cuttings were immediately frozen and stored (−80°C) for later analysis. Due to insufficient DNA yields, only the 60-day incubation data were used for most sites, except for Shizafon 1, where both 60- and 115-day samples were analyzed. Detailed information on sampling periods and locations is available in Supplementary Table ST1.

### 2.2 DNA extraction and sequencing

DNA was extracted using the DNeasy PowerWater Kit (Qiagen) and the PowerSoil® DNA Isolation Kit (Qiagen) following the manufacturer’s protocol. Approximately 0.25 gr of rock cuttings were transferred to the bead tubes provided by the PowerSoil® DNA Isolation Kit. Due to low DNA yields (<5 ng μL^−1^), an additional step was incorporated to improve recovery: after adding the first lysis reagent from the kit, the bead tubes were incubated in a water bath at 70°C for 10 min to enhance cell lysis. The remainder of the protocol followed the manufacturer’s instructions. Limited original rock cuttings from the National Geological Archive restricted the number of replicates due to their scarcity and high value. Library preparation using the NEBNext® Ultra™ IIDNA Library Prep Kit (Cat. No. E7645) and sequencing were performed at the Novogene AIT Genomics facility in Singapore. The nine DNA samples were sequenced using 150 bp paired-end reads on the NovaSeq 6,000 platform, with a read depth of ~15 Gbp per sample.

### 2.3 Assembly, binning, and metabolic predictions

Full-length sequences of the 16S rRNA gene were assembled from the raw metagenomic Illumina reads (this study, and planktonic communities in the respective wells described in Atencio et al., 2024), using phyloFlash v3.4.2 (Gruber-Vodicka et al., 2020), following read mapping to Silva 138 database (outputs available at DOI: 10.6084/m9.figshare.28359185). For metagenomics, Illumina reads were assembled with SPAdes V3.14 (−meta, k = 21,33,66,99,127; Prjibelski et al., 2020), following adapter trimming and error correction with tadpole.sh, using the BBtools suite.^1^ Downstream mapping and binning of metagenome-assembled genomes (MAGs) were performed using DAS Tool, Vamb, Maxbin 2.0, and Metabat2 (Wu et al., 2016; Sieber et al., 2018; Kang et al., 2019; Nissen et al., 2021) within the Atlas V2.11 framework (Kieser et al., 2020), with a genome dereplication nucleotide-identity threshold of 0.975. We curated key metabolic annotations using METABOLIC (Zhou et al., 2022). We used MacSyFinder2 (Néron et al., 2023) with TXSScan (1.1.1) to assess secretion systems and surface appendages, DRAM (Shaffer et al., 2020). KO assignments to evaluate additional genes needed for secretion of extracellular polymeric substances (EPS) and other metabolites useful for biofilm formation, QSAP (Dai et al., 2023) for quorum sensing, and FeGenie (Garber et al., 2020) for potential interactions with iron-bearing minerals. Essential functions were verified using BLAST against NCBI nr/nt and RefSeq databases (Sayers et al., 2022). Tree plotting was performed with iTOL v6.8.1 based on GTDB v2 treeing (Chaumeil et al., 2022). Raw DNA reads and metagenome-assembled genomes were deposited under the NCBI project accession number BioProject PRJNA1149943.

### 2.4 Statistical analyses

Statistical analyses for microbial community structure were performed using R v4.1.1 (R Core Team, 2024), primarily based on the phyloseq package (McMurdie and Holmes, 2013). Hierarchical clustering was performed using pvclust (Suzuki et al., 2019) based on 16S rRNA gene read counts and MAG coverage. Alpha-diversity metrics, including the Shannon index, were calculated for both attached and planktonic community types, and statistical differences were assessed using the Wilcoxon Test (Mann–Whitney U test). This analysis was applied to MAG-based taxonomic profiles. To assess community composition differences, Bray–Curtis dissimilarity was computed, followed by principal coordinate analysis (PCoA) to visualize compositional shifts between attached and planktonic fractions, using both 16S read counts and MAG coverage. Differential analysis of count data was conducted using the package DESeq2 (Love et al., 2014).

## 3 Results

### 3.1 Diversity of the attached microbes

**Figure 3:**
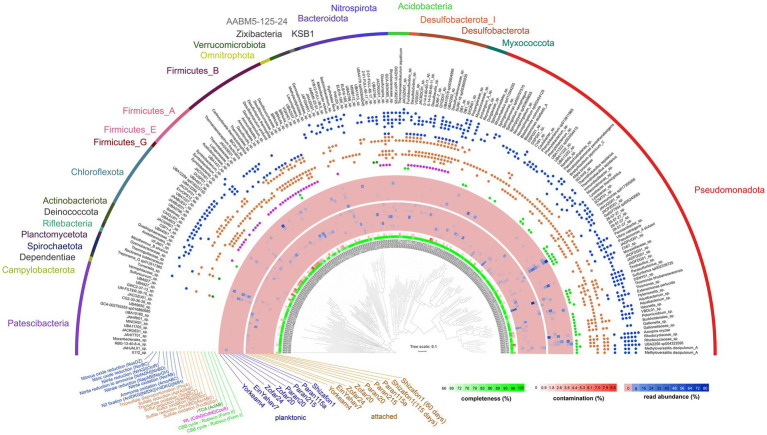
Phylogeny, quality, and read abundance of bacterial metagenome-assembled genomes (MAGs). Key carbon fixation-related functions are shown. Three archaeal MAGs were binned, but excluded from the tree (see Supplementary Table ST3). The relative abundance of MAGs was calculated based on the mapping of reads from both the attached and planktonic datasets. CBB, Calvin–Benson–Bassham cycle; WL, Wood–Ljungdahl pathway.

Metagenomics recovered 194 MAGs from the attached microbial communities (>65% completeness and < 10% contamination, Figure 3; Supplementary Table ST3). These communities were dominated by Pseudomonadota, with Burkholderiales lineages being prominent in the attached communities of all sites (11 to 80% MAG read abundance), except in Ein Yahav 7, Zofar 20, and Zofar 24 (from the JCA, BDA and SCA, respectively). Rhizobiales MAGs were common in the attached fraction of Shizafon 1 (NSA) and Paran 215 (AFA) wells (8–21% read abundance). Other abundant lineages included Desulfotomaculales (Firmicutes) in Paran 20 and Zofar 20 (24 and 20%, respectively) and Desulfarculales (Desulfobacterota, 25%) in Paran 20, whereas Bacteroidales (Bacteroidota, 25%) and Symbiobacteriales (Firmicutes, 13%) dominated Zofar 20. The MAG abundances of these lineages differed between attached and planktonic communities in these wells (Figure 3). However, alpha diversity based on MAG read abundance did not significantly differ between these fractions (Shannon’s Index, Wilcoxon test, *p* = 0.2).

### 3.2 Multiple environmental variables are linked to the attached microbial community composition

Groundwater bicarbonate, nitrate, and hydrogen sulfide concentrations and temperature were linked to the composition of the attached microbial communities (Spearman correlation, *p* < 0.05, Supplementary Figure S3). For example, the read abundances of certain lineages, such as *Thermoanaerobaculum* and GCA-2634385 (family Dissulfurispiraceae), were positively correlated with temperature. Other taxa did not show a statistically significant correlation with temperature but were predominantly found in high-temperature wells. For example, *Dissulfurispira thermophiles* (Umezawa et al., 2021) were exclusively detected in the attached community of the Paran 20 well, in which we recorded the highest temperature of 60°C. Other thermophilic taxa, such as Burkholderiales UBA2250 and *Thermithiobacillus tepidarius* (Wood and Kelly, 1985; Kelly and Wood, 2000; Boden et al., 2016), were present in both Paran 20 and another high-temperature well, Shizafon 1 (> 50°C).

Anaerobic fermenters appeared to be abundant in biofilms on rock cuttings rich in organic content, such as dark charcoal, shale, and bitumen (Zofar 24 and Yorkeam 4 wells). This was particularly evident at the Zofar 24 site (SCA Aquifer), where rock cuttings contained traces of bitumen (Supplementary Table ST1). These conditions supported poorly studied anaerobic microbes, including Bilamarchaeaceae, Micrarchaeota (15%), PJMF01 (12%), UM-FILTER-39-15 Patescibacteria (6%), Desulfurivibrionaceae Desulfobulbia (9%), Melioribacteraceae Bacteroidota (6%), GW2011-AR1 Nanoarchaeota (5%), several Chloroflexota lineages (>5%), and *Rectinema subterraneum* Spirochaetota (3%).

### 3.3 Attached and planktonic microbial communities exhibit distinct taxonomic diversity

**Figure 4:**
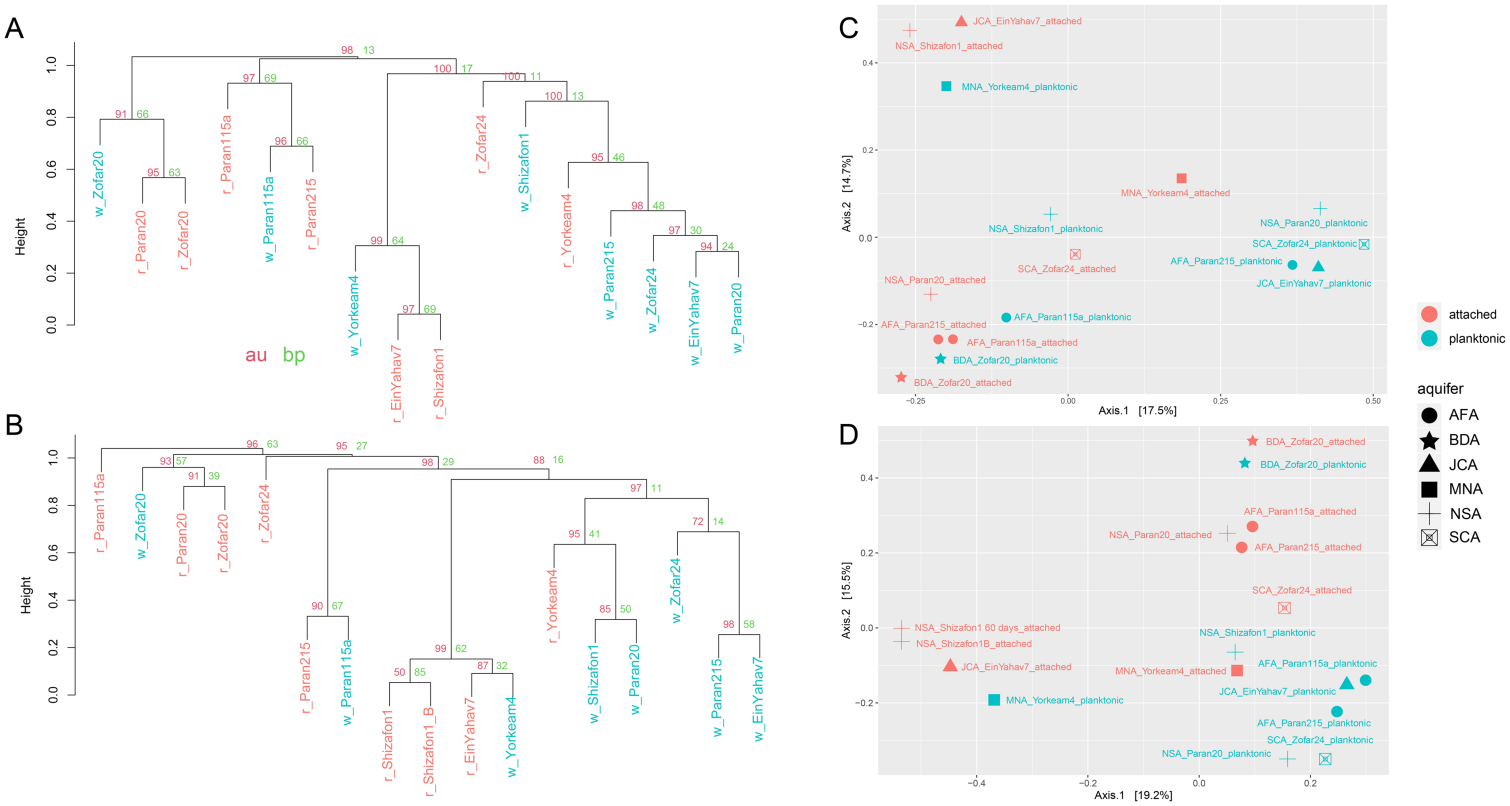
Dissimilarity of planktonic and attached communities in the Negev aquifers. Hierarchical clustering (pvclust, correlation with average clustering method) based on read counts of the assembled 16S rRNA genes **(A)** and the metagenome-assembled genome (MAG) read coverage **(B)**. The attached (red) and planktonic (blue) branches are presented. AU (approximately unbiased *p*-value, red) and BP (bootstrap probability, green) values are shown for each cluster. Principal component analyses based on read counts of the assembled 16S rRNA genes **(C)** and MAG read coverage **(D)**. AFA: Arava Fill and Hazeva aquifers (highly heterogeneous clastic material); SCA: Senonian Chalk & Chert (Mishash formation: cherts, phosphorites, organic-rich carbonates); JCA: Judea Carbonate Group Aquifer (limestone/calcareous); MNA: mixed younger Nubian Sandstone Aquifer (quartzitic sandstone); NSA: Nubian Sandstone Aquifer (quartzitic sandstone); BDA: Brackish Deep Aquifer (quartzitic sandstone).

We observed a partial overlap between the attached and planktonic microbial communities at both the 16S rRNA gene and MAG levels (Supplementary Tables ST4, ST5). The MAG-based analysis revealed that key genera that were common to both community types included *Thiobacillus*, *Sulfuritortus*, *Thermoanaerobaculum*, *Thermithiobacillus*, Halothiobacillaceae 28–57-27, Burkholderiales UBA2250, *Blastomonas*, and *Pseudomonas* A (Supplementary Table ST5). Correlation-based clustering and principal coordinates analysis ordination showed that, in most wells, planktonic and attached communities differed (Figure 4). An exception was Zofar 20, where the planktonic communities showed the highest overlap with the attached ones, largely due to the predominance of *Desulfotomaculum profundi* in both fractions (Figure 4). The MAG read abundance of Pseudomonadota markedly decreased in the attached fraction of Paran 20, Zofar 20, Yorkeam 4, and Zofar 24 wells (Figure 3; Supplementary Table ST5). In contrast, Bacteroidota, Spirochaetota, Chloroflexota, and Nitrospirota were more prevalent in the attached communities at most sample sites (Figure 3). This observation aligns with previous research in the Arava Valley, which reported the predominance of Chloroflexota and Bacteroidota in biofilms from clogged water wells (Laor, 2015).

A detailed examination of specific wells showed that *Thiobacillus* was dominant in the attached communities of Shizafon 1 and Ein Yahav 7 (~40 and 77% of MAG abundance, respectively), constituting a substantial part of the core attached microbial community. However, this pattern was inconsistent across wells, as *Thiobacillus* was more prevalent in the planktonic phase of Yorkeam 4 and Paran 20 wells. In turn, DeSeq2 analyses showed that no taxa were significantly enriched in the attached fraction, while Halothiobacillales 28–57-27 and other Halothiobacillaceae NTUs were enriched in planktonic samples (p-adjusted = 0.05 and 10^−15^, respectively). Other planktonic preferential NTUs included *Candidatus* Nitrosotenuis, *Massilia* sp., and *Microgenomatia* sp. (p-adjusted = 10^−14^, 10^−14^, and 0.03, respectively).

### 3.4 Carbon and energy metabolism of the attached microbes

Metagenomics suggested that the potential to fix carbon is widespread among the biofilm-forming attached aquifer taxa (Figure 3). The form I and II ribulose-bisphosphate carboxylases were widespread among Pseudomonadota, mainly gamma- and alphaproteobacterial, including the prominent *Thiobacillus* MAG 148 (both forms Alfreider et al., 2003). Other organisms, such as *Sulfuricurvum* (Campylobacterota), both MAGs in the Nitrospirota 2-01-FULL-66-17 clade, and the rare Ozeombactericeae lineage, encoded ATP citrate lyase (*aclAB* genes) needed for carbon fixation via the reverse tricarboxylic acid cycle (Hügler et al., 2005). Key genes of the Wood–Ljungdahl pathway (*cdhDE*, *cooS*) were widespread among anaerobes such as Desulfobacterota, Chloroflexota, and Firmicutes. We observed the co-occurrence of CBB and Wood–Ljungdahl pathways in *Desulfotomaculum profundi* MAG 122.

Most of the abovementioned chemoautotrophs carried an array of genes needed for the oxidation of thiosulfate/sulfide via the sulfur-oxidation (SOX) pathway or dissimilatory sulfate reduction (DSR) (Figure 3). Across all wells, we detected 33 MAGs that encoded the SoxBCY components of the Kelly–Friedrich SOX pathway needed for thiosulfate oxidation (Friedrich et al., 2005). The highest read abundances of these MAGs, exceeding 30%, were found in wells Shizafon 1, Ein Yahav 7, Paran 215, and Yorkeam 4 (predominantly Burkholderiales). We identified 56 MAGs featuring DSR and/or reverse DSR metabolism for sulfide oxidation, as indicated by *dsrABD* genes (Figure 3). The *phsA* gene was found in 39 MAGs, indicating genetic potential for thiosulfate disproportionation.

The key terminal oxidases often included co-occurring bd, caa3, and cbb3 types, indicating adaptation to fluctuating oxygen concentrations (Pitcher et al., 2002; Borisov et al., 2011). 21 MAGs encoded the chlorite reduction *cld* gene, enabling chlorite dismutation and microbial oxygen production (Ruff et al., 2023). In turn, nitrate respiration was widespread, as 60 MAGs encoded nitrate reduction to nitrite via transmembrane nitrate reductases (Nar) and periplasmic nitrate reductases (Nap). These included prominent Burkholderiales (e.g., *Thiobacillus* and *Sulfuritortus*), Dissulfurispiraceae, and Caulobacteraceae. Potential for complete denitrification to N₂ was primarily observed in Burkholderiales MAGs, such as *Bradyrhizobium* and UBA2250, while partial denitrification pathways dominated in other taxa.

We found a wealth of genes needed for fermentation, such as those encoding short-chain fatty acid and alcohol conversions (e.g., butyrate, acetate, and lactate). Key acetogenesis proteins, including acetate-CoA ligase, acetate kinase, and phosphate acetyltransferase were encoded by numerous MAGs. Several lineages, including Microgenomatia, ABY1, Paceibacteria, and Clostridia, lacked complete TCA cycles and electron transport chains, suggesting an obligate fermentative lifestyle. We identified several organisms as potential hydrogenogens, often encoding multiple types of [FeFe] hydrogenases (Peters et al., 2015). For example, Symbiobacteriia MAG 67 encoded six different [FeFe] hydrogenases, while *Rectinema subterraneum* MAG 33 and *Desulfotomaculum profundi* MAG 122 carried hydrogenase genes associated with organic matter recycling.

Iron redox enzymes were prevalent in attached microbial communities. In wells Paran 20 and Zofar 20 wells, we identified iron reduction genes in bacteria such as *Carboxydocella thermoautotrophica*. We further identified 20 other MAGs with the potential for iron reduction, spanning various phyla and families. These included Desulfobacterota QYQD01, Meliobacteraceae (genus XYB12-FULL-385), Nitrospirota UBA2194, Dissulfurispiraceae, Burkholderiaceae, and Desulfurivibrionaceae SURF16. These taxa were found across all wells, with the highest abundances in Zofar 24, Paran 20, and Yorkeam 4 (Figure 3). 13 additional MAGs, including those from *Thermoanaerobaculum* and Bryobacteraceae, carried iron-reduction genes. 26 MAGs encoded iron oxidation, including those of *Gallionella*, *Thermoanaerobaculum*, *Aquabacterium*, and Ga007554 species. The abundance of these genes varied across wells, with notable levels in wells Shizafon 1 (15%), Paran 115a (29%), Yorkeam 4 (14%), Paran 20 (7%), and Zofar 20 (8%) (Supplementary Figure S4).

### 3.5 Genetic potential for biofilm formation and interactome in the attached microbes

**Figure 5:**
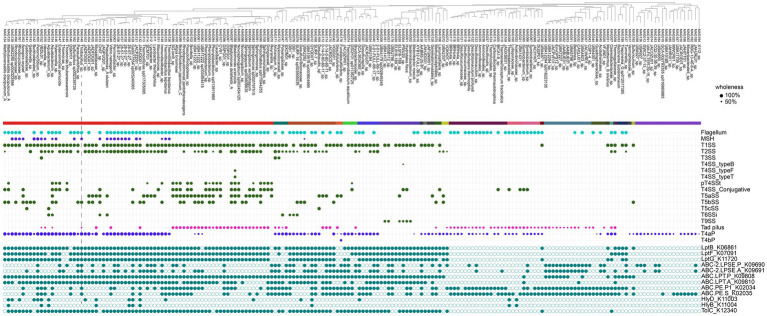
Genetic potential for interactions and biofilm formation in attached bacteria. Macsyfinder results are presented as trait wholeness (% occurrence of canonical features). The absence/presence of features relevant to extracellular polymeric substance (EPS) production is shown at the bottom in cyan.

We found the common occurrence of flagellum gene clusters, especially among Pseudomonadota, Firmicutes, and Desulfuromonadota (Figure 5). Pseudomonadota, including the dominant *Thiobacillus* spp., encoded mannose-sensitive hemagglutinin (MSHA) pili, which are critical for the surface attachment of vibrios (Utada et al., 2014; Zhang et al., 2021). Tad adhesion pili (Pu and Rowe-Magnus, 2018) were common, especially among Alphaproteobacteria MAGs, whereas type 4a pili (Pelicic, 2008) were widespread among various taxa but very few alphaproteobacterial lineages. Multiple secretion systems were found across various attached lineages, indicating a broad range of physical and chemical interactions within these biofilms. We identified numerous genes involved in quorum sensing (Supplementary Figure S5).

We found a wealth of genes related to EPS production and export (Wu et al., 2017; Nuppunen-Puputti et al., 2022) (Figure 5). Type I secretion genes (*hlyB*, *hlyD*, and *tolC*) were found in more than 50% of the MAGs. However, a smaller subset (less than 15%), including taxa such as QYQD01 (Desulfobacterota), Halothiobacillales, and Burkholderiales, carried a complete set of EPS export genes (*tolC*, *hlyB*, and *hlyD*) in the attached fraction. Common *Thiobacillus, Thermithiobacillus*, and *Desulfotomaculum* MAGs possessed the tolC gene but lacked the *hlyB* and *hlyD* genes. We identified genes linked to ABC transporters, which are involved in biofilm formation (ABC.PE.P1/S, ABC-2.LPSE.A) (Hardouin et al., 2022; Dinesh et al., 2023).

## 4 Discussion

Our findings reveal that biofilm formation in deep aquifers is governed by a complex interplay of microbial metabolic strategies, environmental conditions, and geochemical factors. While there is some overlap between planktonic and attached microbial communities (e.g., *Thiobacillus* sp.), biofilms appear to be structured ecological systems rather than random assemblages of taxa. These findings indicate that biofilm formation in these aquifers is shaped by selective pressures related to surface attachment, nutrient availability, and local geochemical gradients. The widespread occurrence of biofilm formation-related traits including mobility, attachment mechanisms, quorum sensing, secretion systems and EPS production highlight the selective pressures associated with biofilm formation, and suggest a broad range of physical and chemical interactions within these biofilms. In turn, groundwater flow dynamics and matrix recolonization processes may influence biofilm development. High flow velocities and water shear forces near boreholes can lead to biofilm detachment, facilitating microbial dispersal and recolonization of new surfaces (Supplementary Figure S6). This process may contribute to the partial overlap between microbial fractions while still allowing for niche differentiation based on environmental conditions.

Temperature and groundwater chemistry appear to play a key role in shaping the composition of attached microbial communities. Thermophilic taxa such as *Dissulfurispira thermophiles*, Burkholderiales UBA2250, and *Thermithiobacillus tepidarius* were predominantly found in high-temperature wells, indicating that certain microbes thrive under extreme thermal conditions. However, some thermophilic lineages were detected in multiple wells without a statistically significant correlation to temperature, suggesting that other environmental factors, such as resource competition and microbial interactions, influence community structure.

Our results suggest that biofilm colonization is strongly influenced by mineral selectivity, particularly in aquifers with iron- and sulfur-rich substrates (Paran 20, Yorkeam 4, Shizafon 1, and Zofar 20; Supplementary Table ST1), following previous findings (Casar et al., 2021; Mullin et al., 2020). Sulfate reduction and sulfur oxidation may fuel microbial autotrophy in these aquifers, given that genes encoding dissimilatory sulfur metabolism were widespread among numerous taxa, such as the prominent *Thiobacillus* sp., the key primary producers in these aquifers (Atencio et al., 2024). Iron cycling may provide alternative energy and shape biofilm composition, as evidenced by the marked occurrence of genes needed for Fe reduction and oxidation. For example, *Carboxydocella thermoautotrophica*, known to reduce Fe(III) minerals such as glauconite via hydrogenogenic carbon monoxide oxidation (Toshchakov et al., 2018), was identified in Zofar 20 and Paran 20 wells, bearing iron-rich minerals limonite, glauconite, and pyrite. Beyond their ecological roles, these taxa (e.g., iron-reducing *Carboxydocella*, iron-oxidizing *Gallionella*, sulfate-reducing *Desulfotomaculum* and sulfur-oxidizing *Thiobacillus*) may contribute to microbial-induced corrosion and well-clogging in groundwater systems, following previous observations in Israeli groundwater systems (Laor, 2015).

In addition to iron and sulfur cycling, organic matter availability appears to play a key role in biofilm formation, particularly in rock cuttings rich in bitumen, shale, or charcoal. The enrichment of fermentative microbes in bitumen-rich wells suggests that anaerobic fermentation is a dominant metabolic strategy in these aquifers, as diverse and abundant microbial populations encoded genes for acetogenesis, short-chain fatty acid, and alcohol metabolism. This finding aligns with previous studies documenting the oxidation of ancient organic matter in subsurface aquifers (Vengosh et al., 2007; Burg et al., 2013). The presence of negative δ^13^C values (Supplementary Table ST2) in dissolved inorganic carbon from Yorkeam 4 further supports the role of organic matter oxidation in shaping microbial metabolism. Fermentation products may serve as substrates for other metabolic processes, contributing to biofilm stability and resilience.

While oxygen is limited (below 0.2 mg L^−1^, except for the two AFA wells, which have approximately 5 mg L^−1^; Supplementary Table ST2), the genetic potential for aerobic respiration appears to be widespread among the attached microbes in the desert aquifers. Together with the discovery of microbial chlorite dismutation *cld* genes in attached microbes, these findings align with those of Ruff et al. (2023), who observed that “dark oxygen” produced through microbial chlorite dismutation supports aerobic metabolisms in anoxic/hypoxic groundwater systems. The persistence of aerobic respiration in ancient groundwater dated up to ~600,000 years provides evidence for the global importance of this process.

In turn, aquifer biofilm microbes exhibit a wealth of adaptation strategies in light of low, and possibly fluctuating oxygen concentrations. These include genes encoding terminal oxidases adapted to varying oxygen concentrations, as well as nitrate respiration enzymes. Thus, both oxygen and nitrate respiration may support energy conservation in these systems. The coexistence of the potential for complete and partial denitrification pathways suggests that nitrogen cycling is likely mediated by cooperative microbial interactions, reinforcing the role of syntrophic relationships within biofilms. We also observed the potential for switching between autotrophic pathways with varying oxygen tolerance: for example, the co-occurrence of CBB and Wood–Ljungdahl pathways in *Desulfotomaculum profundi* MAG 122 (Berg et al., 2022). These transitions may base syntrophic interactions, supporting energy conservation and biogeochemical transformations. Our findings align with previous studies that highlight the ecological importance of facultative anaerobes in deep groundwater environments, where they maintain metabolic flexibility and contribute to redox-dependent processes, biofilm resilience, and biogeochemical cycling (Vick et al., 2019; Anantharaman et al., 2016; Mosley et al., 2022).

## 5 Conclusion

Our study highlights the unique role of the attached communities in the functionality and sustainability of deep groundwater systems. Following environmental selection based on aquifer physicochemical parameters and the mineral composition of settlement substrates, these microbes form biofilms characterized by intricate interactions and an array of metabolic adaptations. We envision that microbial diversity in these aquifers is maintained via the dynamic exchange between attached and planktonic fractions, potentially affected by physical forces near boreholes. Biofouling and redox cycling by these biofilms can play a key role in microbial-induced corrosion and well-clogging, potentially impacting water extraction and treatment systems. Future research asking how these microbial communities influence aquifer stability and infrastructure performance could provide valuable insights into long-term water resource management and groundwater sustainability.

Given these findings, it is essential to enhance sampling strategies focusing on attached and planktonic microbial fractions. Future research should aim to refine these methodologies, particularly by extending observation periods and incorporating environmental conditions that more closely mimic those *in situ* (e.g., conditions undisturbed by pressure and flow). This will provide deeper insights into the role of attached microbial communities and help develop more effective strategies for managing biofouling and other microbial-related challenges in groundwater treatment.

## Data Availability

Raw DNA reads and metagenome-assembled genomes were deposited under the NCBI project accession number BioProject PRJNA1149943. phyloFlash output is available at DOI: 10.6084/m9.figshare.28359185.

## Footnotes

^1^ sourceforge.net/projects/bbmap/
