# Supplementary material for: Site-specific incubations reveal biofilm diversity and functional adaptations in deep, ancient desert aquifers
Source: Front Microbiol. 2025 Mar 21;16:1533115. doi: 10.3389/fmicb.2025.1533115 (PMC11968702; doi:10.3389/fmicb.2025.1533115)
Supplement: Supplementary file 1 [file Data_Sheet_1.pdf]

Supplementary information for:

Site-specific incubations reveal biofilm diversity and functional adaptations in deep, ancient desert aquifers

Betzabe Atencio, Stas Malavin, Maxim Rubin-Blum, Roi Ram, Eilon Adar, Zeev Ronen

Supplementary Note 1

The Nubian Sandstone Aquifer (NSA) contains ancient groundwater, with 81Kr dating suggesting ages ranging from tens to hundreds of thousands of years (Yokochi et al., 2018, 2019; Ram et al., 2020, 2021a). Interestingly, these ages do not consistently increase downstream as expected in a confined system. This irregularity is also reflected in the variable chemical and isotopic composition observed along the eastern flow path, from the southern Negev to the Dead Sea basin (see Figure 2 in the main text).

The irregular pattern of groundwater ages within the NSA prompted Atencio et al. (2024) to conduct a hydrological study examining hydrochemical and isotopic data. Their findings suggested that groundwater mixing, facilitated by intrusions from nearby aquifers, plays a significant role. These intrusions come from aquifers such as the Brackish Deep Aquifer (BDA), the overlying Upper Cretaceous Judea Carbonate Group Aquifer (JCA), the Senonian Chalk and Chert Aquifer (SCA, Mount Scopus group), and the shallower Arava Fill and Hazeva Formation aquifers [AFA] (Figure 2 in the main text) (Atencio et al., 2024). These interconnected aquifers, which have varying lithologies, mineral compositions, and hydrochemistry (Rosenthal et al., 2007; Ram et al., 2020, 2021b; Atencio et al., 2024), offer unique opportunities to explore microbial ecophysiology in deep, ancient groundwater.

Rock cuttings were collected from eight wells, including seven production wells and one observation (artesian) well, scattered across the Negev Desert and the Arava Valley (Figure 2 in the main text). These wells tap into various aquifers (Supplementary Figure S1). Wells such as Shizafon 1 and Paran 20 tap into the Lower Cretaceous Kurnub Group, primarily composed of quartz sandstone with layers of shale, clay, dolomite, pyrite, and other minerals (Rosenthal et al., 1998). Wells such as Yorkeam 4 (Mixed Nubian Aquifer) and Zofar 20 (artesian well, BDA) also screen in front of the Kurnub Group but differ slightly in lithology, with charcoal and bitumen traces found in their rock formations (Supplementary

Table ST1). Zofar 20, in particular, reaches a depth of 1 km and shows signs of intrusion from the BDA (Ram et al., 2020). Nearby, Zofar 24 taps into the shallower Mishash Formation (part of the Mount Scopus Group), which is rich in organic sediments and lies above the JCA (Burg et al., 2013, 2017). The Ein Yahav 7 well taps the JCA, which is mainly composed of limestone and dolomite. Paran 115a and Paran 215 wells access different horizons within the AFA, where sand and gravel dominate (Supplementary Figure S1).

The groundwater in the NSA is predominantly anoxic, characterized by high concentrations of total dissolved solids, sulfate, and dissolved iron, with levels reaching up to ~7000 mg/L, 1000 mg/L, and 16 mg/L, respectively (Supplementary Table ST2 and Supplementary Figure S2) (Rosenthal et al., 1998; Vengosh et al., 2007). In most of the NSA, dissolved iron exists primarily in its reduced form (Fe^{2+}), which rapidly oxidizes and precipitates as iron oxide during water extraction (Vengosh et al., 2007). Iron minerals, commonly found as bands in the NSA's lithology (Rosenthal et al., 1998), further contribute to this process. In addition, many regions of the aquifer exhibit high concentrations of hydrogen sulfide, reaching up to 18 mg/L (Rosenthal et al., 1998; Kamyshny et al., 2008), along with elevated temperatures and pressures, up to 60°C and 144 bars.

Beyond the NSA, most wells also show elevated hydrogen sulfide (HS^-) concentrations (Supplementary Table ST2), except for the Paran 115a and Paran 215 wells in the Karkom graben. These two wells are distinctive, as they contain oxygen levels of approximately 5 mg/L, unlike the anoxic conditions observed in the rest of the wells. Moreover, the highest groundwater temperatures were recorded in the NSA wells, the BDA well, and the Paran 215 well (AFA) (Supplementary Table ST2). All sampled wells exhibited high sulfate concentrations, while nitrate (NO_3^-) was only detected in the AFA wells and Ein Yahav 7 from the JCA. The substantial sulfate, bicarbonate, and ferrous iron concentrations in the samples suggest concurrent terminal electron acceptor processes (Supplementary Figure S2).

Hydrological studies have also detected radioactive elements such as radium, radon, and uranium in groundwater from the Arava Rift Valley (Mazor, 1962; Kronfeld et al., 1992; Minster et al., 2004), suggesting the potential for water radiolysis. This process, occurring as radioactive elements decay, may generate hydrogen - a critical energy source for microbial

activity (Lin et al., 2005; Dzaugis et al., 2016). More detailed discussions of the aquifer's geochemistry and geological characteristics can be found in prior studies (Kroitoru, 1980; Yechieli et al., 1992; Rosenthal et al., 1998; Oren et al., 2004; Vengosh et al., 2007; Burg et al., 2013; Ram et al., 2020, 2021a, 2021b).

References

Atencio, B., Ram, R., Burg, A., Yokochi, R., Yechieli, Y., Purtschert, R., et al. (2024). Investigating the enigma of an irregular groundwater age pattern in a confined, presumed "fossil" complex aquifer through mixing cell flow modeling. *J. Hydrol.* 630:130631. doi: 10.1016/j.jhydrol.2024.130631

Burg, A., Gavrieli, I., and Guttman, J. (2017). Concurrent salinization and development of anoxic conditions in a confined aquifer, Southern Israel. *Groundwater* 55, 183–198.

Burg, A., Zilberbrand, M., and Yechieli, Y. (2013). Radiocarbon variability in groundwater in an extremely arid zone—the Arava Valley, Israel. *Radiocarbon* 55, 963–978.

Dzaugis, M. E., Spivack, A. J., Dunlea, A. G., Murray, R. W., Sylvan, J. B., Moyer, C. L., et al. (2016). Radiolytic hydrogen production in the Subseafloor Basaltic Aquifer. *Front. Microbiol.* 7:76. doi: 10.3389/fmicb.2016.00076

Kamyshny, A., Zilberbrand, M., Ekeltchik, I., Voitsekovski, T., Gun, J., and Lev, O. (2008). Speciation of polysulfides and zerovalent sulfur in sulfide-rich water wells in southern and central Israel. *Aquat. Geochem*. 14, 171–192.

Kroitoru, L. (1980). The hydrogeology of the Nubian Sandstone in southern Israel. [master's thesis] (in Hebrew, English abstract). Tel Aviv, Israel: Tel Aviv University.

Kronfeld, J., Weinberger, G., Yaniv, A., Agami, M., Zafrir, H., Vulcan, U., et al. (1992). Uranium isotope disequilibrium studies and the geohydrology of the Arava Rift Valley, Israel. *Int. J. Radiation Appl. Instrument. Part E Nucl. Geophys*. 6, 535–545.

Lin, L.-H., Slater, G. F., Sherwood Lollar, B., Lacrampe-Couloume, G., and Onstott, T. C. (2005). The yield and isotopic composition of radiolytic H_2, a potential energy source for the deep subsurface biosphere. *Geochim. Cosmochim. Acta* 69, 893–903.

Mazor, E. (1962). Radon and radium content of some Israeli water sources and a hypothesis on underground reservoirs of brines, oils and gases in the Rift Valley. *Geochim. Cosmochim. Acta* 26, 765–786.

Minster, T., Ilani, S., Kronfeld, J., Even, O., and Godfrey-Smith, D. I. (2004). Radium contamination in the Nizzana-1 water well, Negev Desert, Israel. *J. Environ. Radioact*. 71, 261–273.

Oren, O., Yechieli, Y., Böhlke, J. K., and Dody, A. (2004). Contamination of groundwater under cultivated fields in an arid environment, central Arava Valley, Israel. *J. Hydrol.* 290, 312–328.

Ram, R., Burg, A., and Adar, E. M. (2021a). "The Nubian Sandstone Aquifer in the Sinai Peninsula and the Negev Desert," in The Many Facets of Israel's Hydrogeology, eds. U. Kafri, Y. Yechieli

(Switzerland: Springer Nature), 115–141.

Ram, R., Burg, A., Zappala, J. C., Yokochi, R., Yechieli, Y., Purtschert, R., et al. (2020). Identifying recharge processes into a vast "fossil" aquifer based on dynamic groundwater ^{81}Kr age evolution. *J. Hydrol.* 587:124946. doi: 10.1016/j.jhydrol.2020.124946

Ram, R., Purtschert, R., Adar, E. M,. Bishof, M., Jiang, W., Lu, Z. T., et al. (2021b). Controls on the ^{36}Cl/Cl input ratio of paleo-groundwater in arid environments: new evidence from ^{81}Kr/Kr data. *Sci. Total Environ.* 762:144106. doi: 10.1016/j.scitotenv.2020.144106

Rosenthal, E., Jones, B. F., and Weinberger, G. (1998). The chemical evolution of Kurnub Group paleowater in the Sinai–Negev province – a mass balance approach. *Appl. Geochem.* 13, 553–569.

Rosenthal, E., Zilberbrand, M., and Livshitz, Y. (2007). The hydrochemical evolution of brackish groundwater in central and northern Sinai (Egypt) and in the western Negev (Israel). *J. Hydrol.* 337, 294–314.

Vengosh, A., Hening, S., Ganor, J., Mayer, B., Weyhenmeyer, C. E., Bullen, T. D., et al. (2007). New isotopic evidence for the origin of groundwater from the Nubian Sandstone Aquifer in the Negev, Israel. *Appl. Geochem.* 22, 1052–1073.

Yechieli, Y., Starinsky, A., and Rosenthal, E. (1992). Evolution of brackish groundater in a typical arid region: Northern Arava Rift Valley, southern Israel. *Appl. Geochem.* 7, 361–374.

Yokochi, R., Bernier, R., Purtschert, R., Zappala, J. C., Yechieli, Y., Adar, E., et al. (2018). Field degassing as a new sampling method for ^{14}C analyses in old groundwater. *Radiocarbon* 60, 349–366.

Yokochi, R., Ram, R., Zappala, J. C., Jiang, W., Adar, E., Bernier, R., et al. (2019). Radiokrypton unveils dual moisture sources of a deep desert aquifer. *Proc. Natl. Acad. Sci.* 116,16222–16227.

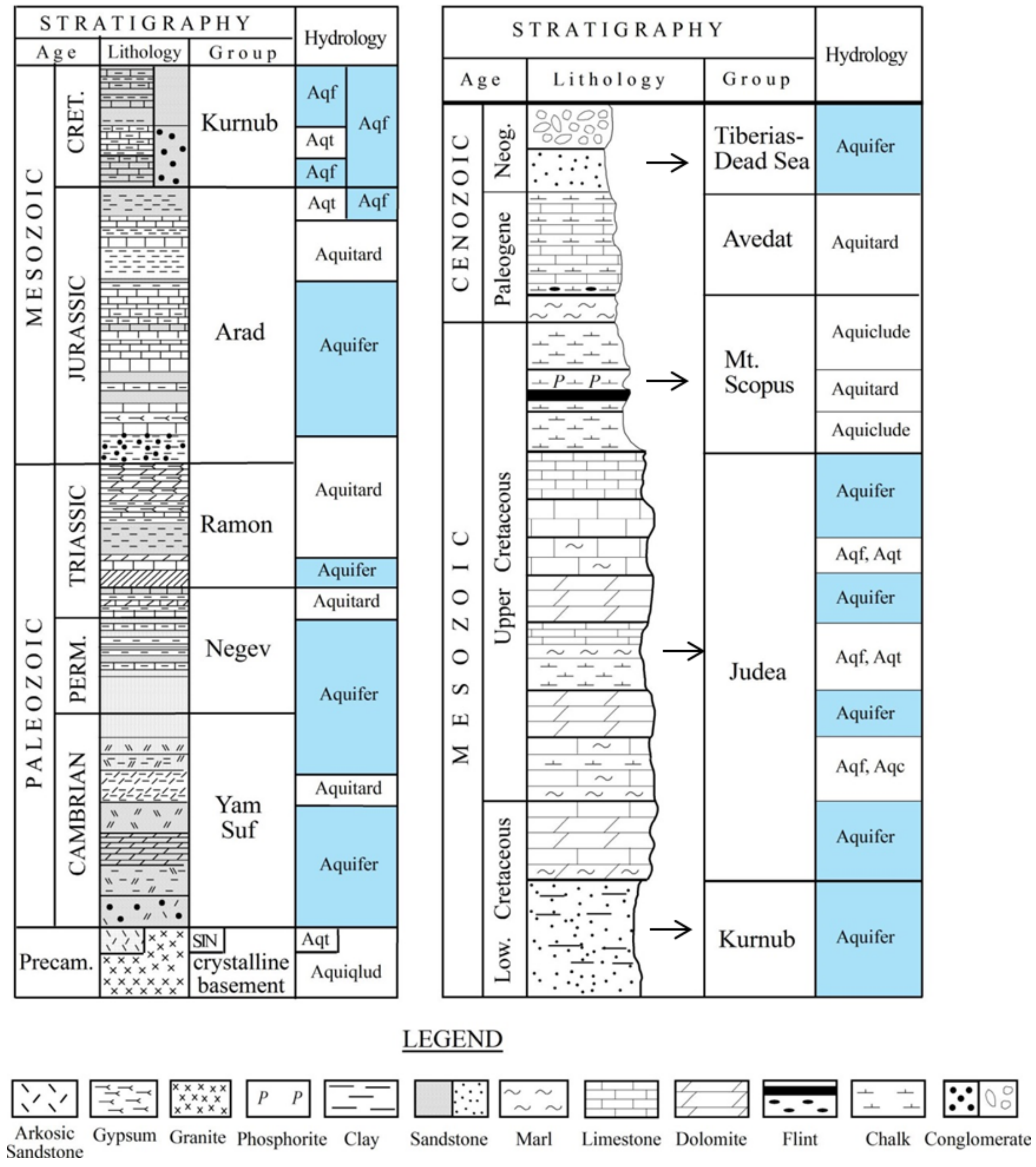

Supplementary Figure S1. Generalized hydrostratigraphic sections for the Negev region. The arrow indicates the approximate location of the sampled sediments (taken from Ram et al., 2021a). The Hazeva Formation and Arava Fill sediments are from the Neogene period.

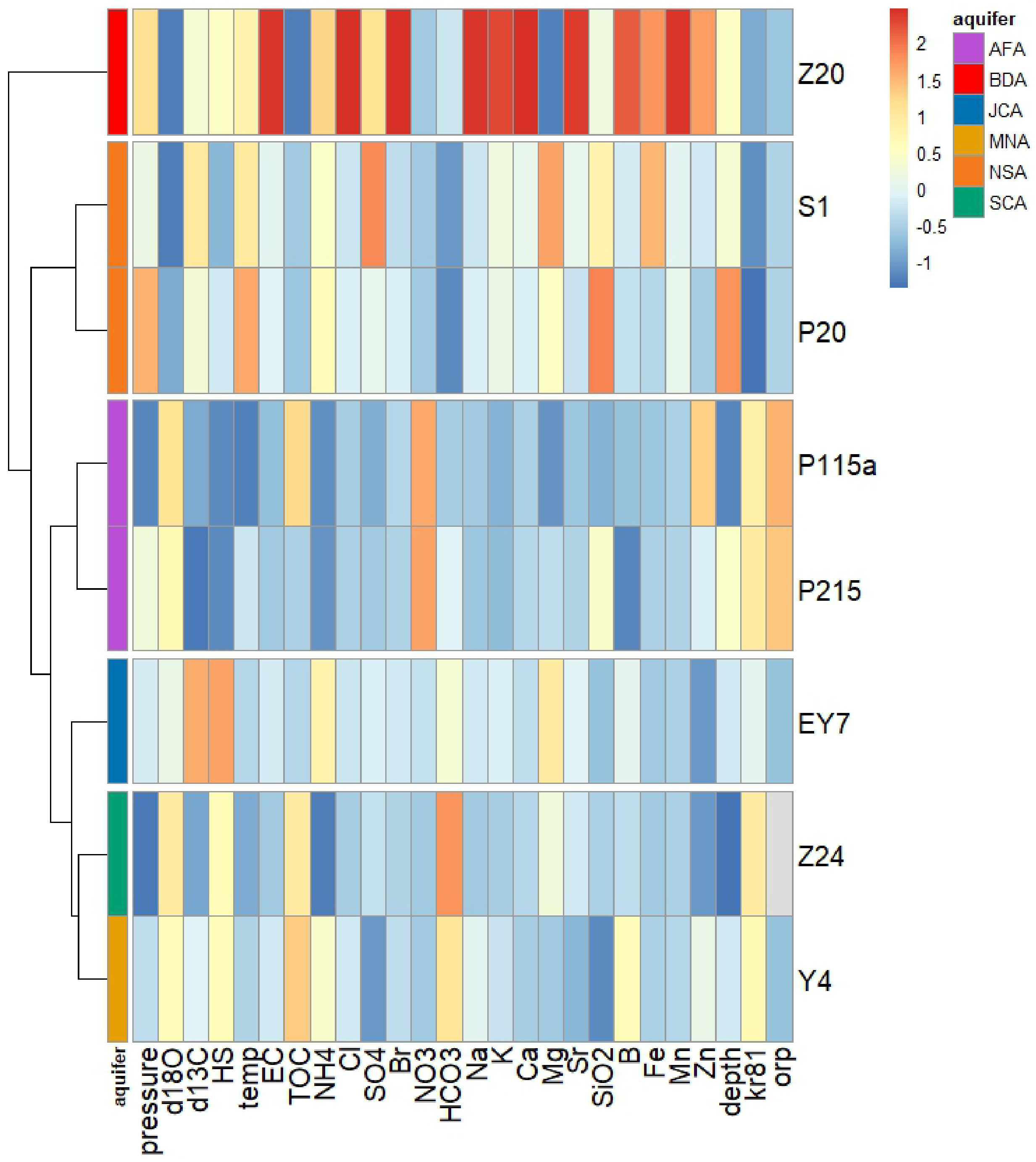

Supplementary Figure S2. A heatmap depicting the hydrochemical and isotopic profiles of the sampled groundwater (data scaled), with shades of red indicating higher values. The wells are grouped using the Ward method of hierarchical clustering.

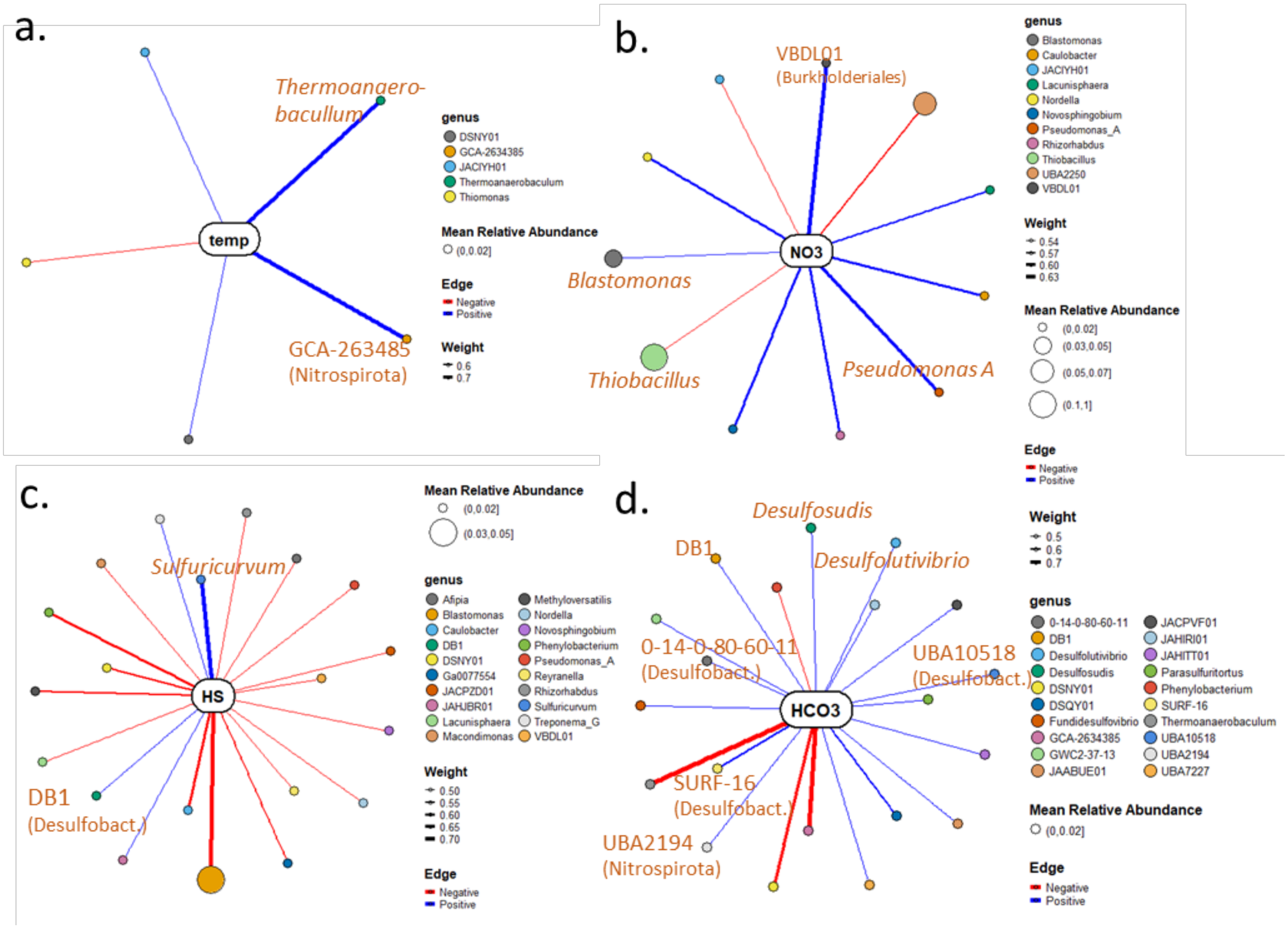

Supplementary Figure S3. Spearman correlation analysis of environmental variables and MAGs on the genus level of attached communities. (a) temperature, (b) nitrate, (c) hydrogen sulfide, and (d) bicarbonate. Red and blue lines represent significant negative and positive associations ($p<0.05$). Higher taxonomic levels are denoted within parentheses in labels.

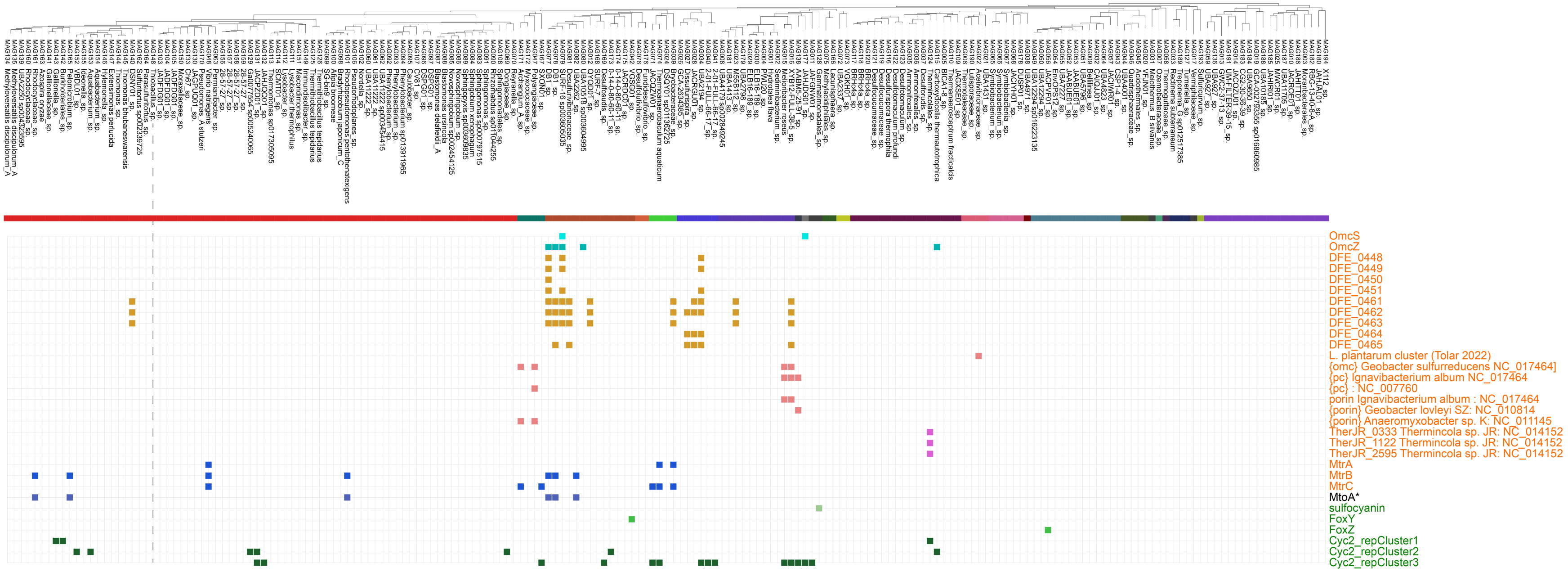

Supplementary Figure S4. Genetic potential for iron reduction (orange) and oxidation (green), or both (black) the attached fraction

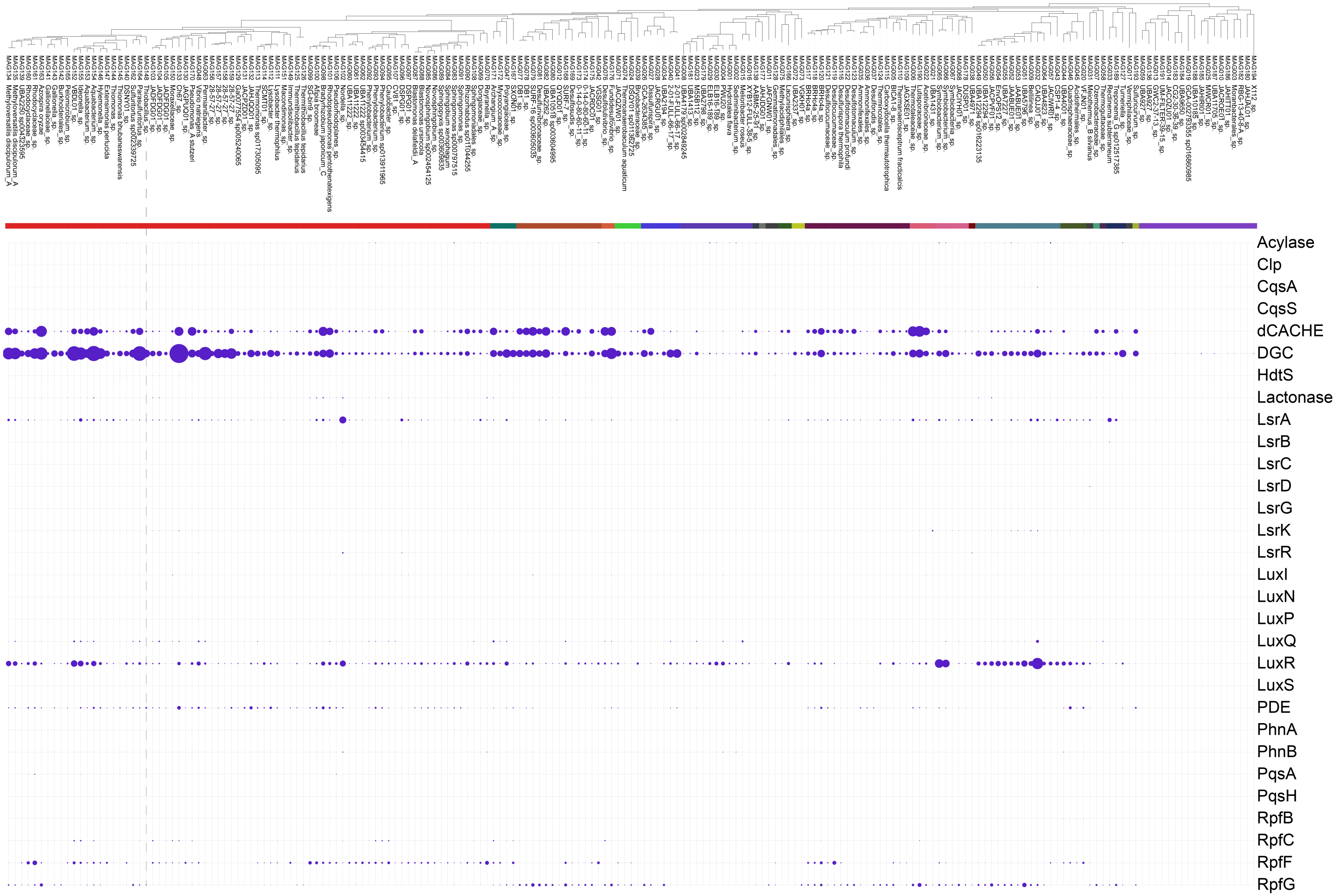

Supplementary Figure S5. The genetic potential for quorum sensing in the attached fraction.

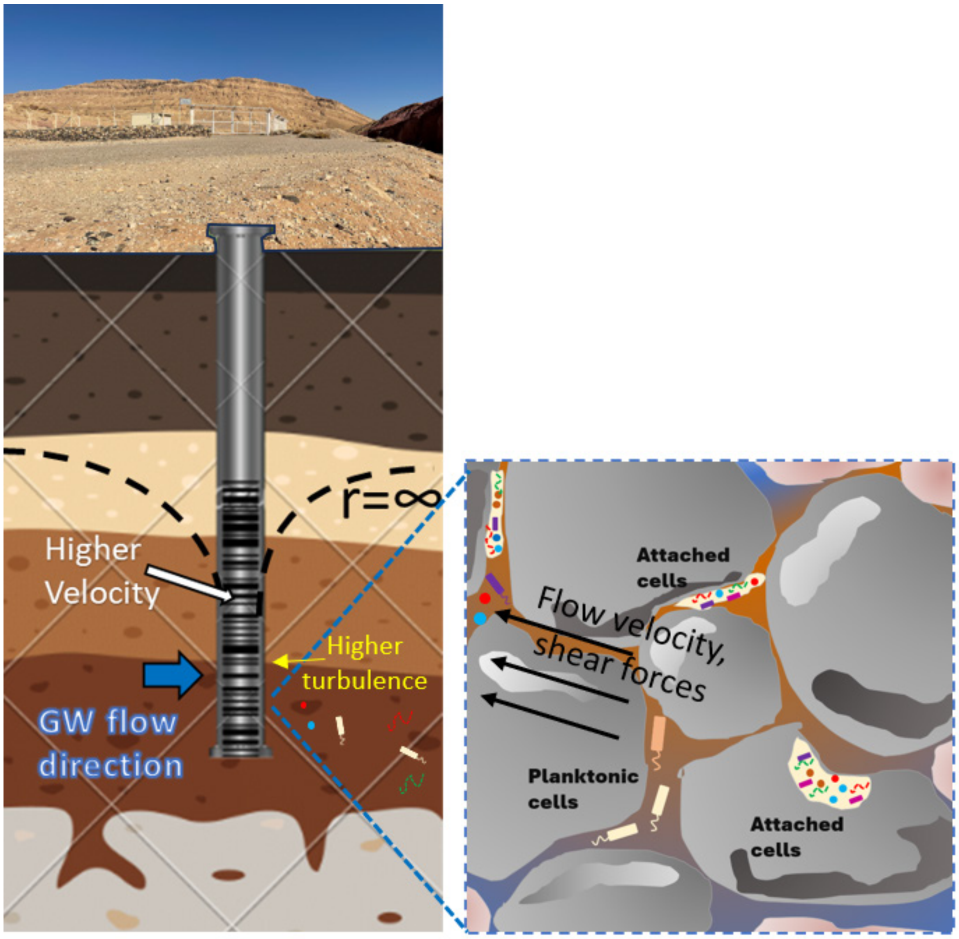

Supplementary Figure S6. Schematic of dynamics of groundwater (GW) pumping, which may cause detachment of cells from the biofilm within the aquifer matrix, where planktonic microbes may transition to a planktonic state.

Supplementary tables (Excel files)

Supplementary Table ST1: Screen intervals and main lithological and mineralogical characteristics of the studied wells. NSA, Nubian Sandstone Aquifer; BDA, Brackish Deep Aquifer; JCA, Judea Group Carbonate Aquifer; SCA, Senonian Chalk & Chert Aquifer; AFA, Arava Fill and Hazeva aquifers. *mbs, meters below the surface (from the Water Authority of Israel). [D] From borehole logs.

Supplementary Table ST2: Hydrochemical, physical, and isotopic characteristics of groundwater of the studied wells. P, pressure; T, temperature; EC; electrical conductivity; bd, below detection levels. [D] Taken from Yokoshi et al. (2019) and Ram et al. (2020). [P] Some values are taken from Yokoshi et al. (2019) and Ram et al. (2021, 2022). *Some values taken from Atencio et al. (2024).

Supplementary Table ST3: Quality, read abundance and taxonomy of metagenome-assembled genomes.

Supplementary Table ST4: Occurrence of attached taxa in planktonic fraction, and vice versa, as determined by cross-mapping of reads on the 16S rRNA sequences determined by phyloFlash.

Supplementary Table ST5: Occurrence of attached taxa in planktonic fraction, and vice versa, as determined by read cross-mapping on the 16S rRNA sequences determined by phyloFlash.

Supplementary Table ST6: Occurrence of attached taxa in planktonic fraction, and vice versa, as determined by read cross-mapping on the metagenome-assembled genomes.
